# Creating a path from the heat shock response to therapeutics of protein-folding diseases: an interview with Rick Morimoto

**DOI:** 10.1242/dmm.014753

**Published:** 2014-01

## Abstract

Rick Morimoto Director of the Rice Institute of Biomedical Research and the Bill and Gayle Cook Professor of Biology at Northwestern University, is renowned for his insights into the heat shock response and its role in protein-folding diseases and aging. As part of *Disease Models & Mechanisms*’ (DMM’s) special series on protein-folding diseases, Rick recalls the karmic events that influenced his career and discusses the importance of invertebrate model systems to reveal the basic mechanisms underlying aging and protein misfolding, as well as efforts to discover therapies to reverse these biological processes.

Richard (Rick) Morimoto was born in Chicago in the USA, just east of Wrigley Field where the Chicago Cubs play baseball. He received an undergraduate degree in Biology from the University of Illinois in Chicago in 1972 and went on to become a PhD student in Murray Rabinowitz’s lab at The University of Chicago, where he helped to map the yeast mitochondrial genome. Rick then joined Matthew Meselson’s lab at Harvard University as a postdoctoral fellow in 1978. His interest in regulation of the heat shock response grew during this time and he, together with collaborators, made the groundbreaking discovery that heat shock genes (now known to encode molecular chaperones) are conserved across a variety of organisms, leading to the cloning of the human *HSP70* gene. In 1982, he set up his own research group at Northwestern University in Evanston, and continued to clone human heat shock genes and the family of regulatory heat shock transcription factors. After combining molecular, cellular and biochemical approaches to understand the regulation of the heat shock response and the function of molecular chaperones, the Morimoto lab adopted the nematode worm *Caenorhabditis elegans* as a model system to explore the fundamental principles of cellular stress responses. In recent years, Rick’s group has provided insights into the role of quality-control systems in the maintenance of proteostasis and how one misfolded protein can initiate a domino effect. In this interview, Rick describes how disruption of these systems underlies the normal process of aging, as well as the pathology of protein-folding diseases. As well as being a research group leader, Rick is one of the founders of Proteostasis Therapeutics Inc., a company that aims to discover and develop therapies for diseases associated with misfolded proteins, such as Alzheimer’s disease, Parkinson’s disease and cystic fibrosis.

**Figure:**
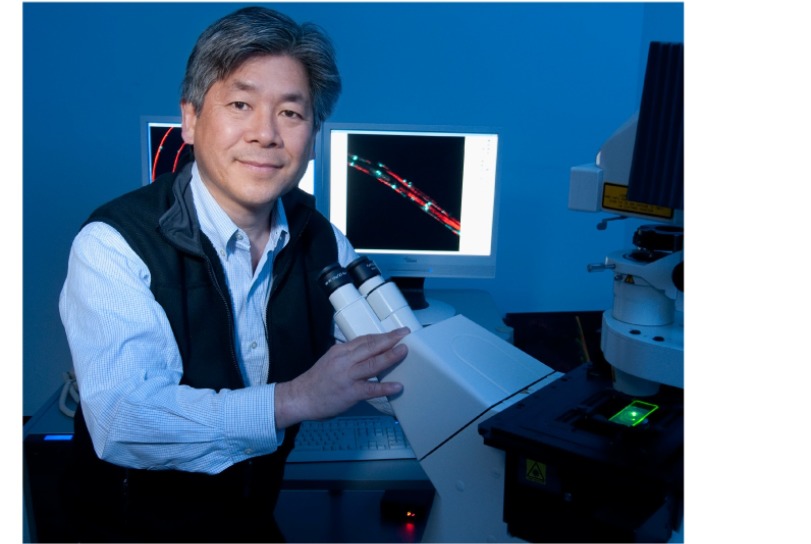


**When did your fascination with science begin? Can you trace your interest to any experiences as a child?**

I’ve always been curious, and science appealed to me from a very young age. I grew up in Chicago and, with enthusiastic support from my parents, had done Science Fair projects from the time I was a young child. When I was 15, I decided I needed to use an electron microscope for a particular project. I had come across a paper, sitting in the stacks of the local library, describing a protist that didn’t fit under traditional categories because it was suspected to have a cellulose wall. This piqued my curiosity and led me to knock on the doors of professors at the University of Illinois at Chicago. Fortunately, I was lucky in that one professor (Howard Buhse) opened his door and let me work in his lab, outside of high school. I worked alongside graduate students who mostly found me an oddity and politely left me alone. Even though I was convinced that I needed an electron microscope to examine the structure of the cell wall, the professor encouraged me to think of other, simpler ways to approach the question. I don’t remember the exact path, but I ended up converting the cellulose into glucose, which is a very easy thing to measure – I used a kit for measuring blood sugar levels in diabetes. Anyway, my experiments turned out pretty well and the protist did have a cellular wall, and for this I received the second place award in biochemistry at the International Science Fair held in Dallas. It seemed pretty neat that I was able to go to Texas to present my findings, and meet other young men and women from all over the world brought together by their interests in science – it was a memorable experience. In those days of working in the laboratories at the university, I remember thinking how wonderful it was to be left alone to be creative with experiments and have fun. I guess I’ve been doing the same thing ever since.

**And that turned out to be the first of many prizes. How did you end up doing a PhD with Murray Rabinowitz?**

As a first year graduate student at The University of Chicago in 1974, I was taking a cell biology course in which we talked about mitochondria and their function and inheritance. The graduate teaching assistant was a fellow named Jim Casey, who happened to be in the lab of Professor Murray Rabinowitz, working on the function of mitochondria in the yeast *Saccharomyces cerevisiae*. Rabinowitz was a very distinguished physician-scientist who had studied at the Rockefeller University and was not a typical MD of that era. We started talking about mtDNA and I asked: “How do we see the DNA? How can we study it?” and he said “Well, that’s what you have to figure out”. So, I joined his group for my PhD and he sent me to Rich Robert’s [Richard J. Roberts] lab at Cold Spring Harbor Laboratory, which, at the time, was one of only two laboratories in the world that had a collection of restriction enzymes. There, I analyzed mtDNA from mitochondria of the so-called grande (wild-type) or petite yeast strains. In the petite strains, deletions were induced in mtDNA using various mutagens but, until the advent of molecular biology, it had not been possible to characterize and localize the deletion sites. By analyzing the DNA restriction pattern, we showed that the ‘petites’ were deletions of large segments of the circular genome. From this, we were able to contribute to the first genomic maps for mtDNA and determine the location of tRNA genes, ribosomal cistrons and components of the mitochondrial-expressed electron transport chains. This formed the basis of my PhD. At a time when molecular biology was just taking off, taking part in this project, going to Cold Spring Harbor and presenting at some of the first yeast meetings was an eye-opening experience.

**What motivated you to work with Matthew Meselson at Harvard University?**

That also came about through what I call karmic events. As I’ve mentioned, as a graduate student I was interested in trying to figure out what mtDNA looks like. I recognized two ways to do this – the first is to use molecular tools (restriction enzymes), which were brand new back then, but the second is to use an electron microscope to visualize the DNA. So, I wandered around The University of Chicago campus to find an electron microscope: looking for electron microscopes seems to be a recurrent theme in my life! Well, I didn’t actually find a microscope, but I did come across Susan Lindquist. Sue had just left Meselson’s lab and was writing her PhD thesis in the laboratory of the cell biologist Hewson Swift. I asked her what she was doing and she showed me some bands on a gel – these were what would become the famous heat shock proteins. We became friends from that point and have been good friends ever since. Two years later, in 1976, Matt Meselson came to The University of Chicago to gain an honorary doctorate and I heard him give the most wonderful talk on the heat shock response in *Drosophila*. The aspect I was most fascinated with, and continues to remain at the forefront of my mind, is how do those little fruit flies know what the temperature is? What is the molecular thermometer? This is a question we’re only beginning to answer now. I didn’t actually meet Matt then, but after his inspirational talk it was arranged that I would go and do a postdoc in his lab at Harvard University.

**Have you worked on invertebrate model systems for most of your research career?**

No, although that is certainly the contemporary impression; we did not use *C. elegans* as a model system until 2000. At Harvard, I did a lot of work on the *Drosophila* heat shock response. I worked closely with people who were also making great progress in this area: Bob Holmgren [now at Northwestern University], Carl Wu [now at Janelia Farm Research Campus and NCI] and Vic Corces [now at Emory University], and I had animated discussions with John Lis [now at Cornell University]. But I kept thinking beyond fruit flies – I wanted to know whether heat shock proteins existed elsewhere. And so I collected DNA from different organisms – silk moth, human, mouse and chicken – and hit them all with restriction enzymes and analyzed the patterns by Southern blotting. For the probe, I radioactively labeled a *Drosophila hsp70* gene using a fantastic method developed by Tom Maniatis. I’ll never forget the moment that I was looking at an X-ray film as it developed and saw bands appearing everywhere. The implications were huge: heat shock genes were not unique to fruit flies; other organisms, including humans, have them too.

Around that time, Tom [Maniatis] had developed the first human DNA library and used it to clone α- and β-globin. I got hold of this library and, together with another postdoc, started working on cloning the human *HSP70* gene. I was still in the Meselson group but moonlighted in Phil Sharp’s [Phillip Allen Sharp] laboratory at MIT. There, I learned a lot about human molecular biology, and shortly afterwards I set up my own lab at Northwestern University, where, for the first phase of my independent research career, I focused on cloning all the human heat shock genes. I had already cloned one at Harvard, and subsequently cloned many other heat shock genes and, shortly thereafter, the heat shock transcription factors. This was strongly inspired by work by Carl Wu, John Lis and Bob Kingston (at Massachusetts General Hospital). Their research highlighted that these heat shock transcription factors were the key stress factors that needed to be understood to find out how and when the heat shock response is used in metazoans.

Towards the end of the ’90s, there were two questions that I was particularly interested in. The first was: how do we translate all our *in vitro* findings about heat shock genes back to an animal? The second was more pragmatic: how are we ever going to study all these heat shock factors? Timing is everything, and around this time a graduate student, Sanjeev Satyal, who had identified a regulator of the heat shock response based on biochemical and molecular approaches, said to me: “There are multiple copies of this heat shock regulator in mice, and there’s only one copy in *C. elegans*. What do you want to do?”. It seemed pretty clear what we should do: we set up a *C. elegans* group. Then came a series of magnificent insights and great papers, including our first story that showed the impact of polyglutamine aggregates on protein homeostasis in *C. elegans*. This instantaneously opened the door to the world of protein conformational diseases and the development of model systems and tools to study these.

**What key features of *C. elegans* make it a good system for studying protein-folding diseases?**

Some people who only work on mammalian systems don’t have a full appreciation of the power of the invertebrate model systems. Having previously used yeast and *Drosophila*, it was relatively easy for us to shift much of the lab from mammalian tissue culture to worms. Once I started using *C. elegans* to characterize the heat shock response, I quickly realized its potential. First off, it’s transparent and by then the green fluorescent protein had been developed, so we could use it to visualize sensory neurons within a defined system. Genetics is of course important, and the fact that the cell lineage was known was a huge advantage. Also, we knew that, when the animal becomes an adult, every cell is post-mitotic. The effects of protein conformational diseases are very dramatic in post-mitotic brain tissue, so this was another advantage. Finally, we had the know-how to be able to specify expression in specific tissue types and at different stages of development.

Not having been trained in neurosciences, it never occurred to me nor was I ever worried that we were working in a model system. Knowing that the heat shock response and molecular chaperones were conserved, I was absolutely convinced that the system could allow us to make discoveries to help build the bigger picture quickly, and this turned out to be the case. And there was a parallel explosion of discoveries across different laboratories, including Sue Lindquist’s remarkable studies in *S. cerevisiae* and Nancy Bonini’s insights using *Drosophila*. We learned so much about how proteins misfold, how they are mis-directed and how clearance properties are altered, and these fundamental ideas have helped us to understand the much more complex, diverse cell populations in humans.

**What does this explosion of discoveries mean for the treatment of protein-folding diseases?**

At the moment, we’re at the transition between discovery and translation. Although there are currently no known small-molecule therapeutics that are mechanistically directed at specific steps in protein homeostasis in humans, there are a number of strategies that show definite promise. FoldRx Pharmaceuticals Inc., the company founded by Sue Lindquist and Jeff Kelly [Jeffrey W. Kelly, The Scripps Research Institute], has led to the development of Tafamidis for the treatment of transthyretin-related hereditary amyloidosis. The small molecule stabilizes the folding of transthyretin, and was shown to work in test-tube biochemistry from model systems to humans, for whom it is now approved in Europe and Japan. This gives us great hope that you can beneficially alter the stability of a protein.

“This has led to the development of a whole new therapeutic strategy that, instead of correcting proteins one at a time, recognizes that the environment of the protein is what has changed”

The challenge is the need to come up with a molecule for every allele, because folding is complicated. Seeking to overcome this, Jeff, Andy Dillin [Andrew Dillin, UC Berkeley] and I established Proteostasis Therapeutics in Cambridge, MA. Together with a group of founders and the chair of our scientific advisory board, Ulrich Hartl, we’re starting to apply the growing body of fundamental insights to the cellular mechanisms that control the folding, stability and functionality of proteins. This has led to the development of a whole new therapeutic strategy that, instead of correcting proteins one at a time, recognizes that the environment of the protein is what has changed, primarily because of the effects of stress and aging. The chronic expression of a misfolded protein then accelerates the damage to quality-control machinery to set off a chain of events that affect the organism on a global level. I like to use the analogy that there is an unexpected increase in the amount of garbage in a city. Initially, the ‘quality-control machinery’ – AKA the garbage trucks and the sanitation workers – manage, but all of a sudden one of the sanitation workers might say, “There’s too much garbage here, I’m not going to pick it all up – I’ll pick the rest on Wednesday” when it’s Monday. You can imagine that it doesn’t take too long for someone to say, “Let that stay an extra week or so”. Over time, the garbage wouldn’t get cleared out, and this would gradually cause problems in the city: people might trip over garbage bags, cars wouldn’t be able to park properly, rats would thrive and the city would smell. This chaos is analogous to the widespread effects of chronic protein misfolding in the cell. However, in cells, a range of alert mechanisms are activated and the unfolded protein response is induced, which helps to protect the cell from damage. When a biological system ages, stress response systems don’t get activated as well, and this is the crucial problem that we are trying to understand in the road to therapy development.

**Do you think that one day we could exploit cellular quality-control systems to slow down aging and extend lifespan?**

We have learned from studies in *C. elegans* and other model organisms that a primary purpose of stress responses is to protect a young, robust, healthy organism in the prime of its reproductive phase. It’s about fitness, and there seems not to be any evolutionary driver to protect the organism when it reaches a phase when fecundity is no longer selected for. Of course, as humans we may not want to accept this – the concept is a little bit too daunting – so there is an obvious interest in applying the knowledge to maintain health and to prevent the disease consequences of human aging. In theory, if we understand the steps of protein homeostasis, we could develop small molecules that increase the activity and robustness of a system as it declines during aging. So, perhaps we could restore the system back to where it was before it started to fail in adulthood. The extent to which you would want to enhance the system is critical, however, and this brings up an interesting question: do the systems fail at the same time in all humans? We can be pretty certain, just by looking at differences in aging across different individuals, that the answer is no – humans do not age at the same rate. There can be a marked discrepancy between chronological age and physiological age. In one of our recent papers, we showed that the collapse of proteostasis is an early event in aging *C. elegans*, but, remarkably, we could re-engineer the system to restore the functional proteome and enhance lifespan. Imagine being able to translate this to humans. Could we monitor the ‘proteostatic health’ of an individual by looking at their mononuclear cells or other accessible tissues, or by using iPSCs, for example? If you observe that the cell’s quality-control systems are struggling, you could think about therapeutically intervening to restore balance. This has implications not only for aging, but also for the early detection of diseases involving misfolded proteins. As we are on the cusp of personalized medicine, the monitoring of proteins within the proteostasis network, which are fundamental to all cellular processes, may provide a chronological history of cellular capacity predictive of the robustness of the quality-control system and therefore risk of age-associated diseases.

“I like to use the analogy that there is an unexpected increase in the amount of garbage in a city…people might trip over garbage bags, cars wouldn’t be able to park properly, rats would thrive and the city would smell. This chaos is analogous to the widespread effects of chronic protein misfolding in the cell”

**You have trained many scientists who have gone on to make great discoveries in this field. What sort of advice do you give to young scientists who come to your lab?**

I give my lab members a tremendous amount of freedom. I have been fortunate as a lot of spectacular young scientists have come to the lab and they always bring fresh and interesting ideas, so I listen to them and we learn from each other. Being able to try new approaches and new ways of thinking is what makes science exciting for everyone. I usually ask potential postdocs to write their fellowships before they arrive in my lab. This helps them to actively engage with the project and the existing lab members before they arrive, so that they feel that they are already part of the team. Of course, I provide a series of ‘hors d’oeuvres’ to inspire them – we have a lot of exciting ideas in the lab, and we use these to encourage a new member to ask questions, take risks and be bold. I also try to make it clear that things don’t always work out as perfectly as you would like, and being able to manage disappointment as well as success is a critical skill.

“…things don’t always work out as perfectly as you would like, and being able to manage disappointment as well as success is a critical skill”

**How do you relax and have fun away from the lab?**

My wife, Joyce, and I enjoy travelling, our garden and our family. We now spend a lot of time visiting our children, Emiko (who is also a molecular biologist) and Kenji (who is engaged in the business world). I’m fortunate because work takes me to many interesting places and provides opportunities to meet new students and colleagues that further stimulates ideas. In the future, some conferences might eventually be replaced by virtual meetings, so I’m making the most of these opportunities while I can. We are also avid gardeners; my wife and I love to work in our perennial garden, and to grow vegetables. We both love the performing arts, and frequently enjoy music and theatre.

